# PRE- AND PERINATAL FACTORS ASSOCIATED WITH WEIGHT GAIN AMONG PRESCHOOL CHILDREN ENROLLED AT DAY CARE CENTERS

**DOI:** 10.1590/1984-0462/2020/38/2019060

**Published:** 2020-01-13

**Authors:** Nykholle Bezerra Almeida, Rísia Cristina Egito de Menezes, Kariny dos Santos Sobral, Jaqueline Fernandes Gomes, Giovana Longo-Silva, Jonas Augusto Cardoso da Silveira

**Affiliations:** a Universidade Federal de Alagoas, Maceió, AL, Brazil.

**Keywords:** Child, preschool, Weight gain, Obesity, Child day care centers, Prenatal care, Breast feeding, Pré-escolar, Ganho de peso, Obesidade, Creches, Cuidado pré-natal, Aleitamento materno

## Abstract

**Objective:**

To identify the factors associated with excessive weight gain in preschool children enrolled at daycare centers in a capital of the Northeast region of Brazil.

**Methods:**

It was a cross-sectional study conducted at the five daycare centers located in the city’s district of most socioeconomic vulnerability. The study included 326 preschool children (17 to 63 months old) from both genders. The dependent variable was the conditional weight gain (CWG), that represents how much a child, according to their gender, deviated from their peers in relation to the expected weight gain, given sample’s birthweight, gender, and age at the survey. Univariate tests (t-test and analysis of variance) were used to compare CWG means according to environmental and biological factors, considering the independent variables with p<0.20 as electable for the multiple linear regression model. In the final model, variables with p<0.05 or that contributed to the model adjustment were kept.

**Results:**

Children’s mean age was 45.4±9.9 months, and 53.4% of the sample consisted of boys. The prevalence of overweight was 7%. In the multivariable linear regression model, it was possible to identify that the following factors were associated with excessive weight gain among preschool children: less than six prenatal care visits (0.36 SD [95%CI 0.13–0.60]), not rooming-in in the postpartum period (0.30 SD [95%CI 0.03–0.58]), and never breastfed (0.44 SD [95%CI 0.06–0.81]).

**Conclusions:**

Inadequate prenatal (appointments) and perinatal care (mother-infant rooming-in and absence of breastfeeding) were associated with excessive weight gain among low-income preschool children.

## RESUMO

**Objetivo:**

Identificar fatores associados com o ganho ponderal excessivo entre pré-escolares de Centros de Educação Infantil (CEIs) em uma capital do Nordeste brasileiro.

**Métodos:**

Estudo transversal realizado em cinco CEIs situados no distrito de maior vulnerabilidade socioeconômica do município. Foram incluídas 326 crianças de ambos os sexos, com idades entre 17 e 63 meses. A variável dependente foi a evolução ponderal condicional (EPC), a qual representa o quanto uma criança desviou do ganho de peso esperado em relação a seus pares, de acordo com o sexo, o peso ao nascer e a idade no inquérito. Análises univariadas (teste *t* de Student ou análise de variância) foram utilizadas para comparar as médias da EPC em função de fatores biológicos e ambientais, considerando como elegíveis para o modelo múltiplo de regressão linear as variáveis independentes com p<0,20; permaneceram no modelo final aquelas que apresentaram p<0,05 ou que contribuíram no ajuste do modelo.

**Resultados:**

A média de idade foi de 45,4±9,9 meses e 53,4% eram meninos. A prevalência de excesso de peso foi de 7%. No modelo múltiplo de regressão linear, identificamos que realizar menos de 6 consultas pré-natal (DP=0,36; IC95% 0,13–0,60), não ter permanecido em alojamento conjunto no pós-parto (DP=0,30; IC95% 0,03–0,58) e nunca ter sido amamentado (DP=0,44; IC95% 0,06–0,81) foram fatores associados com o ganho excessivo de peso entre pré-escolares.

**Conclusões:**

A inadequação dos cuidados pré-natal (consultas) e perinatal (não permanecer em alojamento conjunto e ausência da amamentação) se associaram com o ganho excessivo de peso entre pré-escolares de baixa renda.

## INTRODUCTION

Over the last five decades, Brazilian society has been experiencing a process of food and nutrition transition, with a significant decrease in the prevalence of malnutrition along with an increase in the prevalence of obesity, currently characterized as one of the leading public health problems. Such changes in nutritional profile were so significant that they affected not only adults but also children.^1^

Obesity, which was initially more common in economically developed regions, such as the southeastern and southern regions, was widespread in low-income regions, especially in the Northeast.^1^ A study based on data from national health surveys in the Brazilian population identified, between 1986 and 2006, the prevalence of overweight in preschool children going from 3 to 7.8%; however, the highest prevalence increase occurred in the Northeast Region, where overweight jumped from 1.6 to 7.2% between 1989 and 2006, representing a relative increase of 350%.^2^ In Alagoas (2005–2006), about 9.7% of children under five years of age were obese.^3^

Considering family and society behavioral aspects, excessive weight gain in childhood is related to the adoption of unhealthy eating practices, such as early interruption of exclusive breastfeeding (EBF) through the introduction of infant formulas and/or low nutritional quality infant foods (e.g. ultraprocessed foods and sugary drinks), with subsequent total weaning of breast milk.^4,5^ Such phenomena can stem from issues such as the lack of prenatal guidance or even its poor quality resulting from fragile bond between professionals and individuals. From a broader perspective, the historical role of the food industry in cultural change about infant feeding in early childhood is highlighted, through communication strategies directed at both the general population and health professionals.^5,6^

Therefore, the objective of this study was to evaluate the pattern of weight gain and factors associated with deviations above the expected for age, sex and birth weight among preschool children from a region of high socioeconomic vulnerability in the city of Maceió, Alagoas.

## METHOD

Cross-sectional study conducted at the five day-care centers (DCC) of the seventh health district of Maceió, Alagoas, region of greatest socioeconomic vulnerability of the municipality, entitled “Nutritional situation of children in public day care centers and actions related to food and nutrition in primary care: an intersectoral approach”. All children enrolled in the DCCs aged 17 to 63 months and without physical/motor or intellectual disabilities were considered eligible.

Data collection was made between March and July 2014 by previously trained field teams, with structured and pre-coded questionnaires on biological, dietary, maternal and socioeconomic variables applied to mothers or guardians.

Anthropometry of children under 2 years old or weighing up to 15 kg was made on digital pediatric scale (BP Baby, Filizola, São Paulo, SP, Brazil) and using a 120 cm anthropometric ruler with movable cursor, graduated at 0.5 cm. Children older than 2 years or weighing more than 15 kg were weighed on a digital scale (Plenna, São Paulo, SP, Brazil) and had their height measured on a portable stadiometer (accuracy of 0.1 cm) (Alturaexata, Belo Horizonte, MG, Brazil). Anthropometric indicators — Z-score for weight for age (ZWA) and body mass index for age (ZBMI) — were calculated based on the World Health Organization’s (WHO) growth curve macro.^7^

The child’s weight gain pattern was analyzed based on the conditional weight gain variable (CWG), that is, the standardized residual of gender-specific linear regression models, with the difference between ZWA at birth and at the moment of survey being the dependent variable, and the ZWA of birth weight and current age as independent variable. It represents how much children deviated from the expected weight gain for their age, sex and birth weight, and this variation is expressed as standard deviations (SD). Thus, positive CWG values represent children who gained more weight than expected in relation to their peers. This formulation corrects problems such as the mean regression effect, different ages at the time of research, and collinearity of repeated measures.^5,8^

Biological factors were gender, age, nutritional status and anemia; perinatal factors were birth weight, gestational age, disease at birth and reasons for hospitalization (preventable, non-preventable diseases or absence of diseases). ZBMI was used to sort nutritional status with the following cutoff points: normal weight (<+ 1 SD), risk of overweight (>+1 SD and <+2 SD) and overweight (≥+ 2 SD). Anemia (hemoglobin ≤11 mg/dL) was defined according to the WHO’s recommendations,^9^ and birth disease was characterized as the presence/absence of any birth-related disease.

Maternal variables included: mother’s age, education, number of prenatal appointments, desire for pregnancy, and primiparity. Regarding socioeconomic factors, the following were considered: number of siblings <5 years, monthly per capita income (MPCI) <R$ 70.00, and grant of government assistance. For the categorization of the MPCI, the extreme poverty cutoff at the time of the research^10^ was used. For dietary data, we analyzed breastfeeding at some point in life, time of exclusive breastfeeding (EBF), time of breastfeeding (BF), age of introduction of sugary drinks, and age of introduction of ultraprocessed foods. Finally, the group (nursery I and II /kindergarten I) and the school shift (morning, afternoon, full-time) were also included.

Since the outcome variable had a normal distribution, the CWG means were compared according to the independent variable categories by Student’s t-test or analysis of variance (ANOVA). The independent variables with p<0.20 were considered eligible for the multiple linear regression model, being kept in the model those with p<0.05 or that contributed to the model adjustment. As for the final model, the diagnostic graphs showed that the standardized residuals were distributed randomly and homogeneously on the basis of values predicted by the equation and the independent variables analyzed; Also, no collinearity was observed, as assessed by the variance inflation factor (VIF). Analyses were performed in Stata/SE 15.1 (StataCorp LP, College Station, TX, USA).

This project was funded by the State of Alagoas Research Support Foundation (FAPEAL; Process 60030000692/2013) and approved by the Human Research Ethics Committee of *Universidade Federal do Alagoas* (CAAE 18616313.8.0000.5013). The research was supported by the municipal department of health and by the directors of the DCCs, and only children whose legal guardians signed the informed consent form were included in the sample.

## RESULTS

Among the 366 eligible children, seven were not recruited due to non-signing of the informed consent form by their guardians (n=2) and absence on interview days (n=5). After excluding subjects whose information on birth weight or in the moment of survey was missing (n=33), the sample was formed by 326 children.

Of these, 53.4% were males and 83.1% were older than 36 months, with a mean of 45.4 ± 9.9 months. Respectively, 12.3 and 7.0% of preschool children were at risk of overweight and excess weight, and 44.3% of them were anemic (Table 1). Regarding gestational age, 86.2% had been born at term (≥37 weeks), 92.3% had received breastfeeding at some point in their lives, and only 6.0% had received breastfeeding for 180 days or more. As for the introduction of ultraprocessed foods, 77.3% of mothers reported introducing before 6 months of age (Table 2).

**Table 1:** Characteristics of preschool children attending day care centers in a capital of northeastern Brazil, 2014.

Characteristics	n	%	Mean CWG (SD)	p-value
Gender				
Female	152	46.6	0.00 (1.0)	0.998
Male	174	53.4	0.00 (1.0)	
Age				
<36 months	55	16.9	0.00 (1.0)	0.949
≥36 months	271	83.1	0.00 (1.0)	
Nutritional status				
Normal weight	263	80.7	-0.32 (0.7)	<0.001*
Risk of overweight	40	12.3	0.99 (0.5)	
Overweight	23	7.0	1.96 (0.6)	
Birth weight				
<2.5 kg	30	9.2	0.09 (0.9)	0.574
≥2.5 kg	296	90.8	0.00 (1.0)	
Gestational age				
<37 weeks	45	13.8	0.27 (1.1)	0.050
≥37 weeks	281	86.2	-0.04 (0.9)	
Disease at birth				
Yes	41	12.6	0.13 (0.9)	0.354
No	285	87.4	-0.01 (1.0)	
Reasons for hospitalization				
Never hospitalized	237	72.7	0.00 (0.9)	0.087*
Preventable diseases	66	20.2	-0.14 (1.0)	
Non-preventable diseases	23	7.1	0.39 (1.1)	
Anemia				
Yes	144	44.3	0.03 (1.0)	0.552
No	188	55.7	0.03 (0.9)	
Group				
Maternal I	61	18.7	-0.03 (1.0)	0.953*		
Maternal II	150	46.0	0.01 (1.0)
Jardim I	115	35.3	0.00 (0.9)
School shift				
Morning	178	54.6	0.00 (0.9)	0.486*
Afternoon	86	26.4	0.09 (1.1)	
Full time	62	19.0	-0.10 (1.0)	

CWG: conditional weight gain; SD: standard deviation; *ANOVA.

**Table 2:** Dietary characteristics of preschool children attending day care centers in a capital of northeastern Brazil, 2014.

Characteristics	n	%	Mean CWG (SD)	p-value
Breastfed				
Yes	301	92.3	-0.03 (0.9)	0.033
No	25	7.7	0.40 (0.9)	
Exclusive breastfeeding				
≤30 days	143	47.5	0.04 (1.0)	0.385*
>30 and ≤180 days	140	46.5	-0.11 (0.9)	
>180 days	18	6.0	-0.09 (0.8)	
Breastfeeding				
≤30 days	34	12.5	0.13 (0.9)	0.387*
>30 and ≤120 days	56	20.5	0.08 (1.0)	
>120 days and <180 days	41	15.0	0.11 (1.0)	
>180 days	142	52.0	-0.09 (0.9)	
Age of sugary drinks introduction				
0-6 months	32	9.9	0.12 (1.0)	0.695*
7-12 months	140	43.2	-0.04 (0.9)	
>12 months	152	46.9	0.01 (1.0)	
Age of ultraprocessed foods introduction				
0-6 months	251	77.2	-0.03 (0.9)	0.651*
7-12 months	65	20.0	0.09 (1.0)	
>12 months	9	2.8	0.05 (1.0)	

CWG: conditional weight gain; SD: standard deviation; *ANOVA.

As for mothers, the mean age was 24.8 ± 6.7 years, 23% had gone to less than 6 prenatal consultations, and about 13.8% had had less than four years of study. Socioeconomic data were: 8.4% had income less than or equal to R$ 70, and 76.6% reported receiving a benefit from the government (Table 3).

**Table 3:** Socioeconomic, gestational and perinatal characteristics of mothers of preschool children from day care centers in a capital of northeastern Brazil, 2014.

Characteristics	n	%	Mean CWG (SD)	p-value
Mother’s age				
<21 years	38	11.7	-0.10 (0.8)	0.506
≥21 years	287	88.3	0.01 (1.0)	
Mother’s educational level				
<4 years	45	13.8	0.03 (1.1)	0.824
≥4 years	281	86.2	0.00 (1.0)	
Monthly per capita income				
≤70 BRL	27	8.4	-0.30 (0.9)	0.097
>70 BRL	296	91.6	0.03 (1.0)	
Government Assistance				
Yes	249	76.6	0.00 (1.0)	0.850
No	76	23.4	-0.01 (1.0)	
Planned pregnancy				
Yes	173	53.4	0.03 (1.0)	0.419
No	151	46.6	-0.05 (0.9)	
Prenatal (Number of appointments)				
<6 appointments	75	23.0	0.26 (1.0)	0.008
≥6 appointments	250	77.0	-0.08 (0.9)	
Type of delivery				
Normal	112	34.4	-0.07 (0.9)	0.348
C-section	214	65.6	0.04 (1.0)	
Rooming-in Care				
Yes	274	84.3	-0.05 (0.9)	0.024
No	51	15.7	0.28 (1.0)	
First child				
Yes	108	33.1	-0.02 (0.9)	0.717
No	218	66.9	0.01 (1.0)	
Siblings aging <5 years
None	235	72.1	0.00 (1.0)	0.708*
1 sibling	81	24.8	0.00 (0.8)	
≥2 siblings	10	3.1	0.25 (1.2)	

CWG: conditional weight gain expressed as standard deviation; SD: standard deviation; *ANOVA.

In the univariate analysis, children who had never been breastfed (CWG=0.40±0.9; p=0.033), who had been born with gestational age <37 weeks (CWG=0.27±0.9; p=0.050), whose mothers had <6 prenatal consultations (CWG=0.26±1.0; p=0.008), and whose monthly per capita income was >R$ 70 (CWG=0.03±1.0; p=0.097) had above-average weight gain compared to their peers (Tables 2 and 3). Finally, in the multiple model (Table 4) adjusted for MPCI, mother’s age at birth and child’s height, we found that fewer than six prenatal appointments (SD=0.36; 95%CI 0.13–0,60), having never been breastfed (SD=0.44; 95%CI 0.06–0.81), and not having stayed in joint postpartum rooming (SD=0.30; 95%CI=0.03–0,58) were associated with excessive weight gain.

**Table 4:** Factors associated with conditional weight evolution among preschool children enrolled in day care centers of the seventh sanitary district of a capital of northeastern Brazil, 2014 (n=320).

Characteristics	Reference	β*	95%CI	p-value
Prenatal	<6 appointments	0.36	0.13–0.60	0.003
Rooming-in Care	No	0.30	0.03–0.58	0.032
Breastfed	Never	0.44	0.06–0.81	0.022

*multiple linear regression model adjusted for per capita income below the extreme poverty line, maternal age at birth, and height at the time of the survey; 95%CI: 95% confidence interval.

## DISCUSSION

The objective of this study was to evaluate the factors associated with excessive weight gain among preschoolers in a region of high social vulnerability of a municipality located in the State with the worst performance in the country’s human development index.^11^ We identified that children who had never been breastfed, did not stay in a postpartum room with their mother, and whose mothers had less than six prenatal appointments had greater weight gain than their peers, and the fact that they had never received milk was the factor with most effect magnitude. This paper reinforces the paradigm shift in the relationship between nutritional status and breastfeeding. Overall, even in low-income Brazilian populations, the absence of breastfeeding no longer poses a risk for malnutrition but for overweight.

These findings were interpreted in light of the contextual model of risk factors for obesity in the first thousand days of life, proposed by Baidal et al.^12^ Therefore, from the gaps identified in this systematic review,^12^ the main contributions of this research refer to the effect of community factors (availability and utilization of health services) and mothers’ behavior (dietary style) on children’s weight gain. More specifically, our results reinforce the deleterious effect of the absence of breastfeeding on excessive weight gain, in addition to being one of the few studies that have investigated and established the relationship between pre and perinatal care (consultations and rooming-in care) on the adiposity levels of children.

It is understood that there is a chronological sequence of factors in their contribution to childhood obesity. Starting with prenatal care, smaller number of appointments than the recommendation by the Ministry of Health may have limited the opportunities for health teams to work with pregnant women and prepare them for breastfeeding, also compromising access to information about the benefits and myths of breastfeeding and guidelines for healthy complementary eating.

Another important aspect related to the performance of health services in primary care is the formation of groups of mothers and pregnant women. The low attendance to prenatal consultations may indicate absence of such activities, which are fundamental to build maternal self-efficacy to breastfeed, as it is determined by personal and vicarious experiences, encouragement by others and emotional state.^13^ The fundamental role of joint postpartum rooming is highlighted as a space for strengthening mother-child bond, facilitating the onset of breastfeeding and avoiding early (and often unnecessary) introduction of infant formulas, which can also be classified as ultraprocessed food, and other foods.^14^

It is noteworthy that having stayed in rooming-in care, attended more than six prenatal appointments and never having breastfed the child were factors independently associated with excessive weight gain (afterwards, terms of interaction between factors were tested, but no association was found). Therefore, our hypothesis to justify such associations is related to the construction of positive support networks, both by other women and by professionals involved in the health care of pregnant women.

The importance of prenatal care for the promotion of adequate and healthy child nutrition was demonstrated in a cohort conducted in *Recôncavo da Bahia*, where the absence of prenatal care increased the risk of early ending of exclusive breastfeeding by 173% and the risk of discontinuing breastfeeding by 38%.^15^ In Alagoas, insufficient prenatal consultations was found to increase the risk of introducing ultra-processed foods — hazard ratio (HR) 2.50; 95%CI 1.02–6.16.^16^

Regarding rooming-in care, in the Federal District (capital of Brazil) we found that the prevalence of breastfeeding in the first hour of life was 3.5 times higher among mothers who remained with their children in the postpartum period (83.8%). In a study conducted with low-income families in Curitiba, Paraná, it was observed that this was a protective factor for the duration of breastfeeding (HR=0.37; 95%CI 0.16–0.86),^18^ showing the importance of hospital units to adapt both physically and with protocols to make this practice feasible.

In recent years, the relationship between dietary practices and childhood obesity has been widely explored in the literature, but several aspects still need clarification.^12^ In general, the inconsistencies identified between results can be attributed to the different types of research designs, age used as the cut-off point for discontinuation of breast-feeding, method of assessing food introduction and analytical approach.

A study conducted in municipal day care centers in Recife, Pernambuco, found that children who received exclusive breastfeeding for less than 4 months had a higher prevalence of overweight (22.5%) compared to those who received for 4 months or more (13.5%). Similarly, a study with children aged 12 to 24 months in Bahia showed that exclusive breastfeeding for less than 4 months was also one of the factors associated with overweight – odds ratio (OR) 2.59; 95%CI 1.12–5.99.^20^

Prospective studies initiated in the prenatal or perinatal period identified a similar relationship. Huh et al.^21^ compared children who had never been breastfed or whose total interruption occurred before the fourth month of life with children who had been partially or exclusively breastfed until the fourth month of life. Children in the first group had a 6.3 times higher prevalence of obesity (95%CI 2.3–16.9), but in addition to the prevalence of obesity being higher in this group (∼25%), among those who were maintained in breastfeeding the prevalence was constant (7%), regardless of when solid foods were introduced.

Regarding the pattern of weight gain, a UK cohort found that children under three years old who received solid foods before four months of age had higher weight gain compared to those who continued on breastfeeding (CWG=0.07 SD; 95%CI 0.02–0.11).^4^ In a similar approach, based on nationally representative data, it was found that among Brazilian children (six to 59 months), while each month of maintenance of exclusive breastfeeding had a protective effect on weight gain (CWG = -0.02 SD; 95% CI -0.03–0.00), increasing the daily frequency of sugary drinks had the opposite effect on weight gain (CWG=0.05 SD; 95% CI 0.02-0.08).^5^

One of the theories considered to elucidate the relationship between breastfeeding and obesity addresses metabolic imprinting, that is, nutritional experiences in the first thousand days of life may modulate an individual’s susceptibility to the development of chronic diseases. In the case of obesity, breast milk could influence this process by changing the number and size of adipocytes and promoting neuroendocrine changes in energy balance.^22,23^

On the other hand, another important argument within this discussion concerns the quality of the foods offered during the introduction of complementary feeding. As observed in a previous publication with a similar sample of preschoolers, the period between the third and fifth month of life was found hold the greatest increase in probability of introducing ultra-processed foods in the child’s diet.^16^ In this sense, the protective effect of exclusive breastfeeding would be to delay the introduction of other foods, the predominant and complementary forms limiting the volume of foods offered to the child.

However, it is noteworthy that these hypotheses are not mutually exclusive and, since overweight is established during childhood, there is an increase in the likelihood of maintaining or worsening this condition in the following stages of the life cycle, including development. of other noncommunicable chronic diseases, reduced productive capacity, disability and early death.^24,25^

An important aspect to note is that all variables associated with excessive weight gain in our study are susceptible to modification, both by increasing access to health services and by professional qualification, and improving pre, peri and post-care care infrastructures. Considering the current organization of primary care, such recommendations may be of special value to professionals and health facilities involved in *Rede Cegonha*.

To interpret our results, it is important to consider that the cross-sectional design of the research limits the ability to make causal inferences between exposure and outcome, since one cannot be sure about the exact behavior of children’s weight gain pattern; however, the arguments indicate that there is biological plausibility, coherence and consistency in the scientific literature.

Finally, there are no data available on pre-gestational nutritional status, gestational weight gain or the occurrence of gestational diabetes, factors that could be confounding the association found between weight gain and number of prenatal consultations, from the perspective that pregnant women who were seen more often by physicians could have these conditions controlled. On the other hand, as our outcome variable represents how much the child’s weight gain deviated from peers, the effect of those born heavier as a result of the highlighted conditions would be adjusted.

In this population, characterized by high socioeconomic vulnerability, it can be concluded that children who never received breastfeeding, did not stay in a homestay and whose mothers attended less than six prenatal consultations had a higher than expected pattern of weight gain during childhood when compared to their peers. It is noteworthy that in the current epidemiological scenario of the Brazilian child population, with a significant reduction in the prevalence of malnutrition and an increase in obesity, such a conclusion should not be seen as a recommendation to promote healthy weight gain, especially regarding the introduction of formulas.
